# Effect of high altitude on the pharmacokinetics and pharmacodynamics of valproate in epileptic rats

**DOI:** 10.3389/fphar.2026.1829626

**Published:** 2026-07-01

**Authors:** Xiaojing Zhang, Jiale Song, Hongfang Mu, Rong Wang, Wenbin Li

**Affiliations:** 1 Department of Clinical Pharmacy, The 940th Hospital of Joint Logistic Support Force of Chinese People’s Liberation Army, Lanzhou, China; 2 Department of Pharmacy, The 940th Hospital of Joint Logistic Support Force of Chinese People’s Liberation Army, Lanzhou, China

**Keywords:** epileptic rats, high altitude, pharmacodynamics, pharmacokinetics, valproate

## Abstract

**Background:**

Valproate (VPA) is one of the most widely used drugs for epilepsy. However, it has a narrow therapeutic window and exhibits significant inter-individual variability. Previous studies have suggested that under high altitude conditions, VPA absorption increases and its metabolism slows in healthy rats, indicating that environmental factors can substantially alter its pharmacokinetic (PK) behavior. Nevertheless, it remains unclear how high altitude affect VPA metabolism and efficacy under pathological conditions, such as epilepsy.

**Objective:**

This study aimed to investigate the effects of high altitude on the PK and pharmacodynamics (PD) of VPA in epileptic rats, providing experimental evidence for individualized medication in epilepsy patients rapidly entering high altitude regions.

**Method:**

We prepared the epilepsy model by using the lithium chloride–pilocarpine method. Epileptic rats were randomly assigned to the epileptic + VPA (EV) group and the EV + high altitude (EVH) group for the PK and brain distribution study. VPA concentrations were quantified using a validated liquid chromatography-tandem mass spectrometry (LC-MS/MS) method, and PK parameters were calculated. The expression of P-glycoprotein (P-gp) and hypoxia-inducible factor-1α (HIF-1α) in the blood–brain barrier (BBB) was assessed by Western blot. For the PD study, twenty-four epileptic rats were divided into four groups, including epileptic (E) group, E + high altitude (EH) group, EV group and EVH group. PD effects were evaluated by monitoring seizure scores and the number of seizures. Subsequently, oxidative stress and inflammatory cytokines in brain were measured.

**Results:**

High altitude significantly alters the PK behavior and PD of VPA. Compared with the EV group, EVH group showed lower plasma concentrations, reduced area under the curve, increased clearance, and shorter mean residence time. Meanwhile, the expression of HIF-1α and P-gp in the BBB was significantly up-regulated in the EVH group. PD studies revealed high altitude increased seizure scores and frequency, along with exacerbated oxidative stress and inflammation.

**Conclusion:**

High altitude not only exacerbate seizure severity but also significantly alter the PK and PD of VPA in epileptic rats. This study suggests that epilepsy patients rapidly entering high altitude regions may require an appropriate increase in dosage and enhanced PK/PD monitoring during VPA treatment to ensure clinical efficacy.

## 1 Introduction

Epilepsy is a chronic and recurrent neurological disorder marked by abnormal electrical discharges in the brain, which cause seizures, neuronal damage, motor dysfunction, as well as cognitive and emotional impairment (Li et al., 2021). Approximately 65–70 million people worldwide are epilepsy patients (Pong et al., 2023; Kanner and Bicchi, 2022), with an average annual mortality rate of 1.23% (Xu et al., 2025) and a risk of premature death three times higher than that of the general population (Global, 2025). The prevalence of active epilepsy typically ranges from 4 to 12 cases per 1,000 people, posing a significant burden on patients’ quality of life and risk of premature death, particularly for those experiencing persistent seizures (Thijs et al., 2019). Notably, around 80% of people with epilepsy live in low- and middle-income countries (Fisher et al., 2017). The higher prevalence of epilepsy in these regions compared to high-income countries is likely due to substandard healthcare systems, poor sanitation, inadequate infrastructure, and a higher risk of infections and traumatic brain injuries (Singh and Trevick, 2016).

Epilepsy can last for years or even accompany patients throughout their lives (Hakami, 2021). Currently, drug therapy remains the fundamental and primary treatment approach, aiming to control or reduce seizures within the shortest possible time while avoiding side effects that severely impact quality of life (Thijs et al., 2019). Achieving seizure remission not only lowers morbidity, but also reduces the risk of premature death caused by persistent seizures (Zhou et al., 2026). To date, more than 20 antiseizure medications (ASMs) have been used in clinical practice, with selection primarily based on seizure type (Kanner and Bicchi, 2022). With appropriate drug selection, about 60%–70% of patients can achieve satisfactory control of their seizures (Ng et al., 2025).

Valproate (VPA) is one of the most widely used ASM worldwide (Romoli et al., 2019). It is derived from valeric acid and is a branched-chain short-chain fatty acid compound with a relatively low molecular weight and high-water solubility (Romoli et al., 2019). Although first synthesized as an organic solvent in the late 19th century, its antiepileptic activity remained undiscovered until 1963 (Tomson et al., 2016). It was first approved for epilepsy treatment in 1967 (Tomson et al., 2016). VPA is rapidly absorbed following oral administration with a bioavailability of over 90%, and shows high affinity for plasma proteins, mainly albumin (Kwok et al., 2024). VPA is the primary treatment for various types of epilepsy, including generalized tonic-clonic seizures, myoclonic seizures, and absence seizures (Hakami, 2021). It has been widely used in both adult and child patients. Some hereditary generalized epilepsy can be effectively controlled only with VPA (Hakami, 2021). For seizure control, the clinical dosage range is 0.3–2 g daily (Ornoy et al., 2023), with therapeutic plasma concentrations of 50–100 μg/mL (Ornoy et al., 2023; Hiemke et al., 2018; Cai et al., 2025).

Epilepsy has a higher incidence, prevalence and mortality rate at high altitude than in other areas (Yuxiu et al., 2023). In medicine, high altitude is generally defined as regions above 2,500 m. High altitude environment is characterized by low oxygen, low atmospheric pressure, intense radiation, and cold temperatures (Zhang X. et al., 2024). Hypoxia is the main factor that disrupts normal physiological function (Zhang X. et al., 2024). Hypoxia is a non-specific stressor that induces compensatory adaptive changes in tissue structure, morphology, physiological and biochemical indicators of the body (Mallet et al., 2023). Compared with normoxia, hypoxia affects the activity and expression of drug transporters and metabolic enzymes (Bai et al., 2022; Qiu et al., 2025), thereby interfering with drug absorption, distribution, metabolism, and excretion, and significantly altering PK parameters of the drugs (Liu et al., 2023). For drugs with narrow therapeutic windows, the PK parameters within the body serve as a crucial basis for clinically rational drug administration. Therefore, investigating the changes in the PK characteristics of drugs with narrow therapeutic windows under hypoxia conditions holds significant importance for guiding individualized drug therapy.

VPA is a commonly used ASM worldwide, but it has a narrow therapeutic window. High altitude environment may interfere with the PK, altering the levels of drug exposure in the body and thereby affecting its efficacy while increasing the risk of toxicity. Hu et al. (2022) used a healthy mice model to investigate the effects of a high-altitude environment on the brain-to-blood distribution characteristics of VPA. The results showed that the high‐altitude exposure increased the brain-to-blood distribution of VPA. However, when healthy rats were used as the research model, it was found that the high-altitude exposure instead decreased the brain-to-blood penetration rate of VPA (Lin, 2022). Meanwhile, the PK characteristics of VPA indicated increased absorption and slowed clearance in rats (Lin, 2022). Disease status itself can alter the *in vivo* metabolism of drugs. As an additional stressor, high altitude environment further modulates physiological conditions of the body, thereby affecting drug metabolic characteristics. Based on this, we established an epileptic rat model to systematically investigate the PK and PD of VPA at high altitudes. The objective was to provide evidence-based medication guidance for epilepsy patients living in high altitude areas.

## 2 Materials and methods

### 2.1 Chemicals and reagents

Sodium valproate (Cat: S161023, purity ≥98%), telmisartan (Cat: T129239, purity ≥98%), and pilocarpine hydrochloride (Cat: P129614, purity ≥99%) were provided by Shanghai Aladdin Bio-Chem Technology Co., LTD (Shanghai, China), and telmisartan was used as internal standard (IS). Lithium chloride (Cat: C8381, purity≥ 99%) was provided by Beijing Solarbio Science and Technology Co., Ltd (Beijing, China). Atropine sulfate injection (approval number: H12020382) was provided by Tianjin Jinyao Pharmaceutical Co., Ltd (Tianjin, China). 10% Glucose injection (approval number: H12020022) and 0.9% sodium chloride injection (approval number: H12020024) were provided by Guangdong Otsuka Pharmaceutical Co., Ltd (Guangdong, China). Diazepam injection (specification: 2 mL:10mg, approval number: H50021483) was provided by Southwest Pharmaceutical Co., Ltd (Chongqing, China). Universal tissue fixative (Cat: G1101) was provided by Wuhan Servicebio Technology Co., LTD (Wuhan, China). Reduced glutathione assay kit (GSH, Cat: A006-2-1), superoxide dismutase assay kit (SOD, Cat: A001-3–2), and malondialdehyde assay kit (MDA, Cat: A003-1-2) were provided by Nanjing Jiancheng Bioengineering Institute Co., Ltd (Nanjing, China). Rat interleukin-1β elisa kit (IL-1β, Cat: FXs00283), rat interleukin-6 elisa kit (IL-6, Cat: FXs00271), and rat tumor necrosis factor-α elisa kit (TNF-α, Cat: FXs01560) were provided by Shanghai Fenxi Biotechnology Co., Ltd (Shanghai, China). Primary antibodies of P-gp (Cat: ab170904) and HIF-1α (Cat: 41005) were purchased from Abcam (Cambridge, United Kingdom) and Signalway Antibody (Maryland, United States), respectively.

### 2.2 Instruments and conditions

High-performance liquid chromatography instrument (UFLC-20 A) and triple quadrupole tandem mass spectrometer (API 3200) were provided by Shimadzu (Kyoto, Japan) and AB SCIEX (Redwood City, CA, United States), respectively. A Gemini C18 HPLC column (75 × 3mm, 3µm, Phenomenex) and an acetonitrile-2mmol/L ammonium acetate solution (85:15, V/V) were used as the mobile phase to elute the biological samples. The duration of the process was 3 min, the injection volume was 2 μL, the flow rate was 0.4 mL/min, and the column temperature was 40 °C.

This study used electrospray ionisation sources and negative ion multi-reaction modes for monitoring. The ion spray voltage was −4500 V, the ion source temperature was 400 °C, and the curtain gas was 12 psi. The ion source gas one and the ion source gas two were 45 psi and 20 psi. The declustering potential of VPA and IS were −20 V and −15V, and the collision energies were −10 V and −19 V, respectively. The detection ion pair of VPA and IS was m/z 143.0→143.0 and m/z 513.2→469.2, respectively.

### 2.3 Preparation of solution

#### 2.3.1 Preparation of stock solution

Stock solutions of VPA and telmisartan were prepared in methanol at concentrations of 1.0 and 0.1 mg/mL, respectively. Store them at −20 °C for later use.

#### 2.3.2 Preparation of standard curve samples

##### 2.3.2.1 Plasma standard curve samples

Plasma standard curve working solutions were prepared at concentrations of 1, 5, 10, 50, 100, 300, 600 and 1,200 μg/mL by diluting the VPA stock solution with methanol. A volume of 10 μL of each working solution was added to 90 μL of rat blank plasma to prepare standard curve samples with final concentrations of 0.1, 0.5, 1, 5, 10, 30, 60 and 120 μg/mL.

##### 2.3.2.2 Brain tissue standard curve samples

Brain tissue standard curve working solutions were prepared at concentrations of 0.25, 0.5, 1, 2.5, 5, 10, 50 and 100 μg/mL by diluting the VPA stock solution with methanol. A volume of 10 μL of each working solution was added to 90 μL of rat blank brain tissue to prepare standard curve samples with final concentrations of 0.025, 0.05, 0.1, 0.25, 0.5, 1, 5 and 10 μg/mL.

#### 2.3.3 Preparation of quality control (QC) samples

##### 2.3.3.1 Plasma QC samples

Plasma QC working solutions were prepared at concentrations of 1, 10, 100 and 1,000 μg/mL by diluting the VPA stock solution with methanol. A volume of 10 μL of QC working solution was added to 90 μL of blank plasma to prepare plasma QC samples with final concentrations of 0.1, 1, 10, and 100 μg/mL.

##### 2.3.3.2 Brain tissue QC samples

Brain tissue QC working solutions were prepared at concentrations of 0.5, 5 and 50 μg/mL by diluting the VPA stock solution with methanol. A volume of 10 μL of QC working solution was added to 90 μL of blank brain tissue to prepare brain tissue QC samples with final concentrations of 0.05, 0.5, and 5 μg/mL.

### 2.4 Preparation of sample

#### 2.4.1 Preparation of plasma samples

A total of 200 μL of pre-cooled acetonitrile was added to 50 μL rat plasma for protein precipitation. The mixture was centrifuged at 10,000 g for 15 min, and the supernatant was transferred into a clean tube. The organic solvent was removed using a vacuum centrifuge concentrator (Christ RVC 2–25, Osterode, Germany). The residue was resuspended in 80 μL of mobile phase (containing 5 μg/mL IS), and centrifuged at 10,000 g for 15 min to collect the supernatant for analysis.

#### 2.4.2 Preparation of brain tissue samples

Rat brain tissue was weighed and homogenized using pre-cooled 0.9% sodium chloride injection (W/V = 1/2). 50 μL brain tissue homogenate was mixed with 250 μL of pre-cooled acetonitrile and vortexed for 1 min. Subsequently, the mixture was centrifuged at 10,000 g for 15 min, and the organic solvent was removed using a vacuum centrifuge concentrator (Christ RVC 2–25, Osterode, Germany). The residue was resuspended in 50 μL of mobile phase (containing 1 μg/mL IS), and centrifuged at 10,000 g for 15 min to collect the supernatant for analysis.

### 2.5 Method validation

#### 2.5.1 Specificity

Rat plasma and brain tissue samples (blank or containing VPA and IS) were processed according to the method described in Section 2.4. The processed samples were then subjected to LC-MS/MS for analysis.

#### 2.5.2 Linearity

50 μL of plasma or brain tissue standard curve sample were operated according to the method described in Section 2.4. Standard curves for VPA in plasma and brain tissue were established using weighted least squares to perform linear regression. The x-axis represented drug concentration, and the y-axis represented the peak area ratio of the analyte to the IS.

#### 2.5.3 Precision and accuracy

VPA QC samples in plasma and brain tissue were prepared according to the Section 2.4. Six replicates were prepared for each concentration to assess intra-day and inter-day variations. QC samples were measured on the same day and for three consecutive days to evaluate the intra-day precision, inter-day precision and accuracy. Precision was measured by calculating the relative standard deviation (RSD), and accuracy was determined by comparing the measured value with the true value.

#### 2.5.4 Extraction recovery and matrix effect

VPA QC samples in plasma and brain tissue were prepared according to the Section 2.4 and analyzed. The measured concentration of VPA was recorded as A1. In addition, separated blank rat plasma or blank brain tissue homogenate were prepared using the same method. VPA was then added to the supernatant to prepare plasma or brain tissue sample solutions of identical concentration. The measured concentration of VPA was recorded as A2. At the same time, purified water was used to replace the rat plasma or brain tissue homogenate to prepare VPA solution of the same concentration. The IS working solution was then added and the concentration of VPA was recorded as A3. The extraction recovery and matrix effect were calculated according to the Equations 1, 2:

$$\text{Extraction recovery }\left(\%\right)=A1/A2\times 100\%$$ (1)

$$\text{Matrix effect }\left(\%\right)=A2/A3\times 100\%$$ (2)

#### 2.5.5 Stability

VPA QC samples were prepared according to Section 2.4 to investigate the stability of VPA in plasma and brain tissue samples under different storage conditions. We also investigated the stability of the mobile phase in QC samples stored at room temperature over different time. Three replicates for each concentration. Stabilities were expressed as the concentrations after different operations to the concentration at time zero.

### 2.6 Experimental design

#### 2.6.1 Animals

SPF-grade male Wistar rats, 2 months old, weighing (200 ± 20) g, purchased from Shandong Pengyue Laboratory Animal Technology Co., Ltd (Shandong, China). This study was approved by the Ethics Committee of 940th Hospital of Joint Logistic Support Force of Chinese People’s Liberation Army (approval number: 2022KYLL204, approval date: 18 Oct 2022). All animal experiments were conducted in accordance with the relevant animal ethics guidelines and regulations.

#### 2.6.2 Development of an epilepsy model

Lithium chloride-pilocarpine method was used to establish an epilepsy model in this study (Wu et al., 2025; Di Liberto et al., 2018; Xiong et al., 2025). Rats received an intraperitoneal injection of lithium chloride solution (127 mg/kg) 1 day prior to the injection of pilocarpine solution. A 30 mg/kg pilocarpine solution was injected intraperitone-ally 30 min after the administration of 1 mg/kg atropine sulfate solution. Observed seizures according to the Racine standard. If grade IV or higher seizures were not observed within 30 min, an additional 10 mg/kg pilocarpine solution was injected. This process was repeated until the target seizure grade was achieved. In this study, each rat received up to three additional injections of pilocarpine, with a maximum cumulative dose of 60 mg/kg. After 60 min of continuous epileptic seizure, diazepam injection (5 mg/kg) was intraperitoneally injected to terminate the seizure. Rats that were successfully modelled were given a 10% glucose solution until they were returned to a normal diet and drinking water. During the modeling process, a total of 42 rats were excluded from the study. 30 rats were excluded because they did not develop status epilepticus, and a further 12 were excluded because they died after modelling and could not therefore proceed to subsequent experiments.

#### 2.6.3 Hematoxylin-eosin (HE) staining and nissl staining

The brain tissues of normal or epileptic rats were fixed with universal tissue fixative, embedded, embedded in paraffin wax, dewaxed to water and stained using HE or Nissl staining. The tissues were then sealed and examined for pathological changes.

#### 2.6.4 Animal grouping

Epileptic rats were randomly assigned to be housed under either plain or high-altitude conditions. Each group was then subdivided into two treatment subgroups: an epileptic group (E for plain and EH for high altitude) and a group treated with VPA (EV for plain and EVH for high altitude). Experiments under plain condition were conducted at the Animal Laboratory of 940th Hospital of Joint Logistic Support Force of Chinese People’s Liberation Army in Lanzhou of Gansu Province (an altitude of 1,500 m, oxygen content 18.55%, temperature 20∼25 °C, humidity 50∼60%). Rats assigned to the high-altitude condition were quickly transported to the high-altitude laboratory in Yushu Autonomous Prefecture of Qinghai Province (an altitude of 4,010 m, oxygen content 12.70%, temperature 20∼25 °C, humidity 50∼60%) via temperature-controlled freight vehicles combined with air transport. The entire transport process was completed within 24 h and the relevant experiment began after the rats had undergone acute hypoxia for 3 days. The oxygen level remained stable at 12.70% throughout the experiment.

#### 2.6.5 PK studies

Rats in the EV and EVH groups were fasted for 12 h and then orally administered 94.5 mg/kg VPA (n = 6 per group). Blood samples (0.5 mL) were collected into heparin-coated EP tubes from the jugular vein cannulation before and at 5, 10, 20, 45 min and 1, 2, 4, 6, 8, 12, 24 h after administration. After each blood collection, an equal volume of sterile physiological saline was immediately infused via the jugular vein cannula. Plasma samples were collected by centrifuging the blood samples at 3,000 g for 10 min at 4 °C. Following the final blood collection, the rat’s brain tissue was removed. Biological samples were stored at −80 °C until analysis. PK data were analyzed using WinNonlin software (version 8.1). Non-compartmental model was selected to estimate the PK parameters of VPA in both groups of rats. The PK parameters were calculated according to the Equations 3‐9:

$$\text{Half}‐\text{life }\left(t_{1/2}\right)=\ln\left(2\right)\;/\;\lambda_{z}$$ (3)

$${\text{AUC}}_{\left(0‐\infty \right)}={\text{AUC}}_{\left(0‐t\right)}+\text{Clast}/\;\lambda_{z}$$ (4)

$$V=\text{Dose}/\left(\;\lambda_{z}\times {\text{AUC}}_{\left(0‐\infty \right)}\right)$$ (5)

$$\text{CL}=\text{Dose}/{\text{AUC}}_{\left(0‐\infty \right)}$$ (6)

$${\text{MRT}}_{\left(0‐t\right)}={\text{AUMC}}_{\left(0‐t\right)}/{\text{AUC}}_{\left(0‐t\right)}$$ (7)

$${\text{MRT}}_{\left(0‐\infty \right)}={\text{AUMC}}_{\left(0‐\infty \right)}/{\text{AUC}}_{\left(0‐\infty \right)}$$ (8)

$${\text{AUMC}}_{\left(0‐\infty \right)}={\text{AUMC}}_{\left(0‐t\right)}+\left(\text{Tlast}\times \text{Clast}/\;\lambda_{z}\right)+\left(\text{Clast}/\;\lambda_{z}^{2}\right)$$ (9)

Clast: observed concentration corresponding to Tlast, λ_z_: terminal elimination rate constant, estimated by linear regression of time vs*.* log-concentration, AUC_(0-t)_: area under the curve from the time of dosing to the time of the last measurable concentration, AUMC_(0-t)_: area under the moment curve from the time of dosing to the last measurable concentration.

#### 2.6.6 Brain distribution studies

Rats in the EV and EVH groups were fasted for 12 h and then orally administered 94.5 mg/kg VPA (n = 30 per group). Blood and brain tissue samples were collected at 5, 45 min and 1, 2, 24 h after administration (6 rats at each time point). Plasma samples were collected by centrifuging the blood samples at 3,000 g for 10 min at 4 °C. Plasma and brain tissues were stored at −80 °C until analysis.

#### 2.6.7 Western blotting analysis

As previously described, the isolation of brain microvessels was carried out (Elfakhri et al., 2018; Abdallah et al., 2021). In brief, brain tissues were homogenized in ice-cold Dulbecco’s Phosphate-Buffered Saline (DPBS, Servicebio, Wuhan, China), and one volume of 30% Polysucrose 400 (Aladdin, Shanghai, China) was added. Following centrifugation of the homogenate at 8,000 g for 10 min, the pellet was resuspended in ice-cold DPBS supplemented with 1% BSA. The suspension was then passed through a glass bead column to isolate microvessels that adhered to the beads.

Western blot was used to determine the expression of P-gp and HIF-1α in isolated microvessels. The total protein concentration for each sample was determined using a BCA protein assay kit (Cat: PC0020, Solarbio, Beijing, China). Protein samples were separated by 8% SDS-PAGE gel electrophoresis, transferred onto membranes, blocked with 5% skim milk, and washed with 1×TBST. Subsequently, the membranes were incubated with primary antibodies against P-gp (1:5,000), HIF-1α (1:1,000), and β-actin (1:50,000) overnight at 4 °C. After washing, the membranes were incubated with goat anti-rabbit secondary antibody (1:5,000) for 2 h at room temperature, followed by washing with 1×TBST. Target protein bands were detected using an ultra-sensitive ECL chemiluminescence assay kit (Beyotime, Shanghai, China) and imaged by autoradiography. The gray values of the protein bands were scanned using Quantity One software. β-actin was used as the loading control. The relative expression level of the target proteins (P-gp and HIF-1α) was calculated as the ratio of the gray intensity of each target protein band to that of the β-actin band for the same sample.

#### 2.6.8 PD studies

Rats in the EV and EVH groups were administered VPA (94.5 mg/kg) once daily for 7 days, whereas those in the E and EH groups received saline (n = 6 per group). Seizure scores and the number of seizures were observed for 90 min following the administration on the seventh day. Blood and brain tissue samples were collected 24 h later. Plasma samples were collected by centrifuging the blood samples at 3,000 g for 10 min at 4 °C. Biological samples were stored at −80 °C until analysis.

Rat brain tissue samples were weighed and homogenized using pre-cooled saline (1/9, W/V). The mixture was centrifuged at 1,000 g for 10 min and the supernatant was used as 10% homogenate supernatant for assay. The levels of MDA, GSH, SOD, IL- 1β, IL-6, and TNF-α in brain tissue were strictly determined in accordance with the assay kit requirements. Each sample was measured in triplicate.

### 2.7 Statistical analysis

In this study, all experimental results were expressed as mean ± standard deviation (mean ± SD). A two-tailed Student’s t-test was used to analyze VPA concentrations in plasma and brain tissue after 7 days of continuous administration. One-way ANOVA was used to analyze the results regarding rat seizures, oxidative stress, and inflammation. Two-way ANOVA was applied to analyze the results of the mean plasma concentration-time curves and brain tissue distribution. Bonferroni test was used as the *post hoc* test for multiple comparisons. P < 0.05 was considered to be statistically significant.

## 3 Results

### 3.1 Method validation

#### 3.1.1 Specificity

**FIGURE 1:**
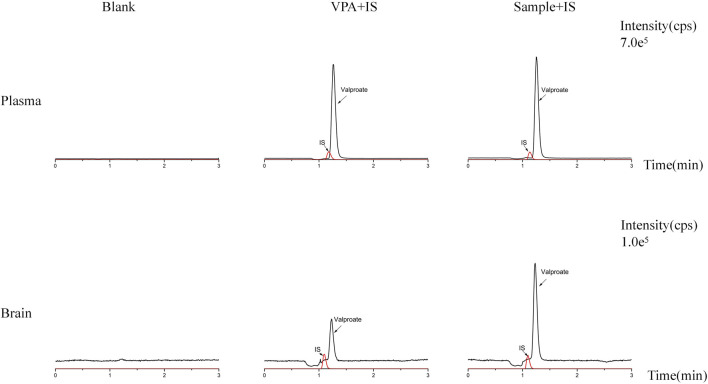
Typical chromatograms of VPA and IS in rat plasma and brain tissue samples.

Typical chromatograms of VPA and IS in rat plasma and brain tissue are shown in Figure 1. The presence of endogenous substances in plasma and brain tissue did not significantly interfere with the determination of the target analyte.

#### 3.1.2 Linearity

The standard curve equations of VPA in rat plasma and brain tissue samples were y = 0.9749x+0.5452 (*R* ^2^ = 0.9,976) and y = 1.0145x+0.0054 (R^2^ = 0.9,998), respectively. This result indicated that VPA had a good linearity in plasma and brain tissue.

#### 3.1.3 Precision and accuracy

The intra-day and inter-day RSD of VPA in rat plasma and brain tissue were both less than 15%. The intra-day and inter-day accuracies in plasma were 95.5%–101.8% and 96.7%–100.7%, respectively. The intra-day and inter-day accuracies in brain tissue were 99.5%–105.5% and 97.7%–104.5%, respectively. This result met the methodological requirements for biological samples (Table 1).

**TABLE 1:** Intra-day and inter-day precision and accuracy of VPA (n = 6).

Sample type	Concentration (μg/mL)	Intra-day precision	Intra-day accuracy	Inter-day precision	Inter-day accuracy (%)
		(RSD, %)	(%)	(RSD, %)	(%)
Plasma	0.1	11.0	100.3	2.5	97.5
	1	8.0	95.5	1.1	96.7
	10	6.2	99.2	0.7	98.6
	100	9.1	101.8	1.0	100.7
Brain	0.05	5.2	105.5	4.9	100.3
	0.5	5.3	104.4	0.7	104.5
	5	2.0	99.5	2.1	97.7

#### 3.1.4 Extraction recoveries and matrix effects

The extraction recoveries and matrix effects of VPA are shown in Table 2. In rat plasma samples, the extraction recoveries and matrix effects were 90.5%–93.7% and 94.4%–96.5%, respectively. In brain tissue samples, the respective values were 93.3%–95.5% and 92.9%–97.9%, respectively. These results indicated good extraction recovery of VPA in rat plasma and brain tissue, with no obvious matrix effect from the biological samples.

**TABLE 2:** Extraction recoveries and matrix effects of VPA.

Sample type	Concentration	Extraction recovery (%)		Matrix effect (%)	
	(μg/mL)	Average (%)	RSD (%)	Average (%)	RSD (%)
Plasma	0.1	90.5	4.1	95.8	8.3
	1	92.9	6.6	96.5	6.6
	10	93.5	3.3	94.4	2.8
	100	93.7	4.4	95.3	1.7
Brain	0.05	94.1	7.4	93.1	2.8
	0.5	93.3	6.5	92.9	3.8
	5	95.5	4.9	97.9	1.2

#### 3.1.5 Stability

This study investigated the stability of VPA under different storage conditions. The results are shown in Table 3. The long-term (−80 °C for 20 days) stability of VPA in plasma and brain tissue samples was between 92.3% and 103.7%. After three freeze-thaw cycles, the stability was between 91.4% and 100.3%, and the short-term (room temperature for 24 h) stability was between 92.2% and 101.3%. In addition, the stability in the mobile phase was also evaluated after storage at room temperature for 8, 12 and 24 h. The results showed that the recovery in the mobile phase was between 90.0% and 109.6% (Table 3). The results demonstrated that VPA was stable during both sample preparation and analysis.

**TABLE 3:** Stabilities of VPA under different storage conditions.

Sample type	Concentration	In plasma (%)			In 85% acetonitrile solution (%)		
	(μg/mL)	−80 °C for 20 days	Freezing-thaw 3 cycles	Room temperature for 24 h	Room temperature for different time		
					8 h	12 h	24 h
Plasma	0.1	97.6 ± 11.5	91.7 ± 7.0	92.2 ± 9.3	99.7 ± 11.6	92.7 ± 10.9	94.0 ± 7.4
	1	103.7 ± 10.7	98.3 ± 7.1	101.3 ± 5.9	97.0 ± 5.7	92.3 ± 4.4	90.0 ± 6.7
	10	92.3 ± 4.1	92.7 ± 1.6	99.9 ± 1.4	95.0 ± 8.4	91.7 ± 7.3	94.7 ± 9.0
	100	96.2 ± 3.1	95.9 ± 4.2	93.4 ± 10.1	97.5 ± 0.5	96.2 ± 2.9	94.2 ± 2.7
Brain	0.05	94.9 ± 5.3	91.4 ± 4.7	96.7 ± 8.6	96.6 ± 3.6	94.9 ± 6.6	96.3 ± 12.0
	0.5	99.8 ± 9.7	100.3 ± 5.8	101.1 ± 3.1	106.6 ± 7.5	109.6 ± 6.2	100.5 ± 8.5
	5	96.7 ± 5.0	97.0 ± 1.9	98.8 ± 2.9	101.2 ± 3.0	101.6 ± 4.0	99.1 ± 4.9

Data are presented as mean ± SD (n = 3).

In conclusion, the VPA analysis method established in this study exhibited good linearity. Its precision, accuracy, extraction recovery, matrix effect and stability all met the methodological requirements for the analysis of biological samples, and it can be used for subsequent research.

### 3.2 Brain tissue pathological changes

**FIGURE 2:**
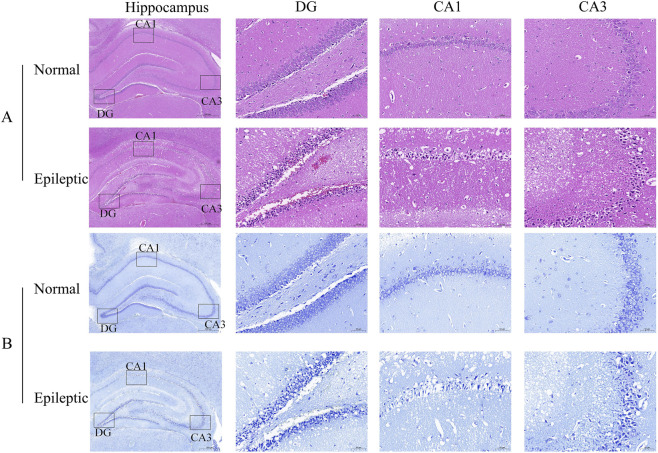
**(A)** HE staining and **(B)** Nissl staining in the hippocampus of normal and epileptic rats. The panoramic images of the whole hippocampus were taken at 3×, whereas the magnified images of DG, CA1, and CA3 subregions were captured at 20×.

A successfully induced epileptic state was defined by the following two criteria: 1) the development of grade IV-V seizures after pilocarpine injection, with behaviors ranging from tail erection and jumping to generalized convulsions and loss of posture, and 2) seizure activity persisting for more than 60 min. Figure 2 showed the pathological damage in the brain tissue of epileptic rats.

As revealed by HE and Nissl staining, neurons in the DG, CA1 and CA3 regions of the hippocampus in normal rats were numerous and neatly arranged. They exhibited intact morphology with well-defined boundaries, and their cytoplasm contained abundant Nissl bodies. In epileptic rats, the DG, CA1 and CA3 regions of the hippocampus exhibited severe damage, as indicated by a significant decrease in the number of neurons, their disordered arrangement, pyknotic nuclei and deepened staining. Additionally, there was cytoplasmic oedema, increased cell volume and a reduction in Nissl bodies.

### 3.3 *In vivo* PK studies results

**FIGURE 3:**
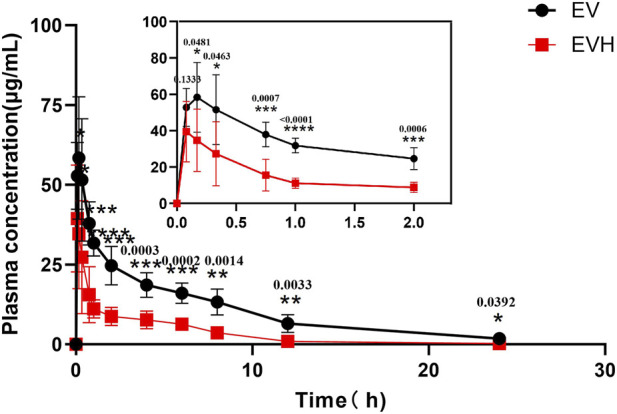
Mean plasma concentration-time curves of VPA after oral administration in the EV and EVH groups. The inset shows the data for the first 2 h. Data are presented as mean ± SD (n = 6). *P < 0.05, **P < 0.01, ***P < 0.001 and ****P < 0.0001.

The PK of VPA in the two groups of rats were determined using a validated analytical approach. The mean plasma concentration-time curves were presented in Figure 3. After drug administration, the plasma concentration of VPA in the EVH group was significantly lower than in the EV group. This indicated that the high altitude environment significantly alters the *in vivo* behaviour of VPA.

The PK parameters of VPA in the two groups of rats were simulated using a non-compartmental model. As shown in Table 4, compared with the EV group, the peak concentration (C_max_) of VPA in EVH group decreased (40.48 ± 17.00 μg/mL vs. 60.92 ± 18.23 μg/mL), and the half-life (t_1/2_) was shortened (3.57 ± 0.34 h vs. 6.48 ± 3.63 h). However, these differences were not statistically significant (P > 0.05). In contrast, three key parameters showed significant changes in EVH group. The mean residence time (MRT) was shorter (4.31 ± 1.04 h vs. 6.18 ± 0.71 h, P < 0.01), the clearance (CL) was faster (1.11 ± 0.26 L/h/kg vs. 0.33 ± 0.06 L/h/kg, P < 0.0001), and the area under the curve (AUC) was reduced (88.51 ± 23.34 h*μg/mL vs. 267.97 ± 50.85 h*μg/mL, P < 0.0001). These changes collectively indicated reduced systemic exposure and enhanced drug elimination.

**TABLE 4:** PK parameters of VPA in the EV and EVH groups.

Parameters	Units	EV group	EVH group	P value	95% confidence interval
T_max_	h	0.14 ± 0.05	0.11 ± 0.05	0.2897	−0.08979 to 0.02979
C_max_	μg/mL	60.90 ± 18.23	40.48 ± 17.00	0.0726	−43.09 to 2.256
t_1/2_	h	6.48 ± 3.63	3.57 ± 0.34	0.0792	−6.228 to 0.4082
V	L/kg	3.10 ± 1.67	5.74 ± 1.50^*^	0.0164	0.5979 to 4.685
CL	L/h/kg	0.33 ± 0.06	1.11 ± 0.26^****^	<0.0001	0.5355 to 1.011
MRT_(0-t)_	h	6.18 ± 0.71	4.31 ± 1.04^**^	0.0045	−3.013 to −0.7269
MRT_(0-)_	h	8.54 ± 2.97	4.64 ± 1.07^*^	0.0126	−6.780 to −1.037
AUC_(0-t)_	h*μg/mL	267.97 ± 50.85	88.51 ± 23.34^****^	<0.0001	−230.4 to −128.6
AUC_(0-)_	h*μg/mL	290.49 ± 51.22	89.61 ± 23.21^****^	<0.0001	−252.0 to −149.7

Data are presented as mean ± SD (n = 6).

* P < 0.05.

** P < 0.01.

****P < 0.0001.

### 3.4 Brain distribution results

**FIGURE 4:**
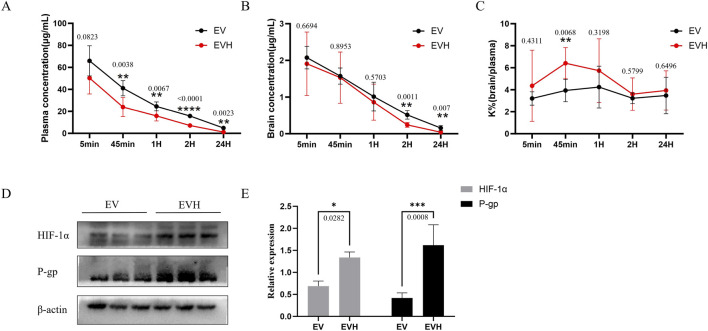
**(A)** Plasma concentration of VPA (n = 6) **(B)** brain concentration of VPA (n = 6) **(C)** Brain/plasma ratio (K%) of VPA (n = 6) **(D,E)** expression of related proteins in BBB (n = 3). Data are presented as mean ± SD. *P < 0.05, **P < 0.01, ***P < 0.001 and ****P < 0.0001.

The concentration of VPA in the brain tissue of both groups exhibited a time-dependent decline. Compared with the EV group, the EVH group exhibited lower VPA concentrations in plasma (Figure 4A). In brain tissue, VPA concentrations in the EVH group were significantly reduced by 53.1% and 72.9% at 2 h and 24 h after administration, respectively (Figure 4B). Furthermore, the brain-to-plasma ratio was consistently higher in the EVH group, with a significant increase of 62.9% observed at 45 min (Figure 4C).

The protein expression levels in the BBB of both rat groups are shown in Figures 4D,E. The expression of HIF-1α and P-gp in the EVH group was 1.94 times and 3.85 times that in the EV group, respectively.

### 3.5 *In vivo* PD studies results

**FIGURE 5:**
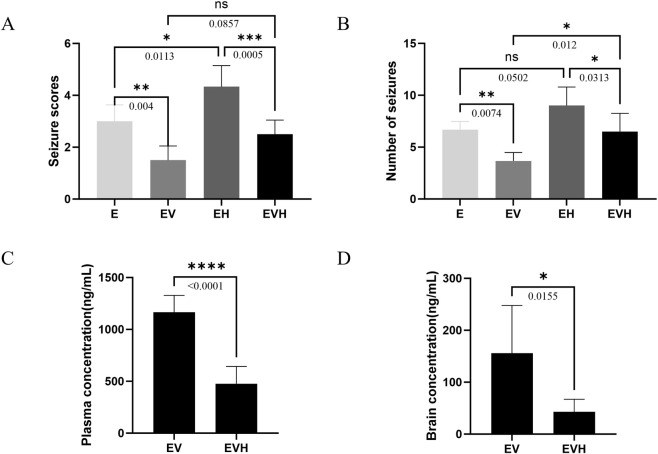
**(A)** Seizure scores **(B)** number of seizures **(C)** plasma concentration of VPA, and **(D)** brain concentration of VPA. Data are presented as mean ± SD (n = 6). *P < 0.05, **P < 0.01, ***P < 0.001, and ****P < 0.0001.

As shown in Figures 5A,B, during the first 90 min of observation on day 7 of VPA treatment, the high altitude significantly increased the number of seizures in the EVH group compared with the EV group (P < 0.05). The seizure scores were also increased in the EVH group, but this difference did not reach statistical significance (P > 0.05). Within 90 min of treatment, both groups of rats exhibited significantly reduced seizure scores and the number of seizures. Overall, compared to the pretreatment baseline, VPA reduced the seizure scores and the number of seizures by 52.6% (1.50 ± 0.55 vs*.* 3.17 ± 0.41) and 50.0% (3.33 ± 0.52 vs*.* 6.67 ± 0.82), respectively, during the first 90 min of treatment in the plain environment. By contrast, VPA was less effective in high altitude environment, reducing seizure scores and the number of seizures by 38.5% (2.67 ± 0.82 vs*.* 4.33 ± 0.82) and 27.8% (6.50 ± 1.76 vs*.* 9.00 ± 1.79), respectively. Additionally, we measured the concentration of VPA in plasma and brain tissue 7 days after administration. Compared with the EV group, the plasma and brain concentrations in EVH group were significantly reduced by 59.1% (477.33 ± 151.40 ng/mL vs*.* 1,166.17 ± 147.31 ng/mL) and 72.6% (42.77 ± 22.31 ng/mL vs*.* 155.85 ± 83.94 ng/mL), respectively (Figures 5C,D).

**FIGURE 6:**
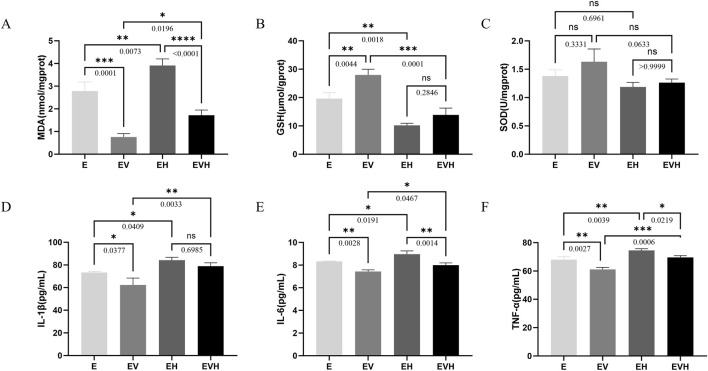
**(A)** Malondialdehyde (MDA) **(B)** reduced glutathione (GSH) **(C)** superoxide dismutase (SOD) **(D)** interleukin-1β (IL-1β) **(E)** interleukin-6 (IL-6), and **(F)** tumor necrosis factor-α (TNF-α) in brain tissues of epileptic rats. Data are presented as mean ± SD (n = 3). *P < 0.05, **P < 0.01, ***P < 0.001, and ****P < 0.0001.

The levels of oxidative stress and inflammatory in the brain tissue of epileptic rats are presented in Figure 6. Compared to the E group, the EH group exhibited markedly exacerbated oxidative stress and inflammatory damage in the brain tissue. Both of these were significantly alleviated by VPA treatment, with the EV group demonstrating greater improvement than the EVH group.

## 4 Discussion

VPA remains a widely used and practical choice for epilepsy treatment, due to its broad range of indications, proven efficacy, low cost, and relatively favorable adverse effect profile compared to other first-generation ASMs (Ying et al., 2024). However, VPA possesses a narrow therapeutic window (50–100 μg/mL) and exhibits significant inter-individual variability (Li et al., 2023). Subtherapeutic levels (<50 μg/mL) may lead to poor seizure control, while supratherapeutic levels (>100 μg/mL) increase the risk of serious adverse reactions, including tremor, confusion, hepatotoxicity, and hyperammonemia (Gziut and Thanacoody, 2025). Therapeutic drug monitoring is essential in clinical practice to maintain concentrations within the safe and effective range. Importantly, high altitude environment may alter the PK and toxicological characteristics of ASMs, potentially elevating the risk of adverse reactions (Yuxiu et al., 2023). Consequently, a systematic investigation into the effects of hypoxia on the PK of VPA is of significant clinical importance. In this study, a rapid and sensitive LC-MS/MS method was developed to quantify VPA in epileptic rats, enabling the investigation of differences in PK behaviour between animals maintained at plain and high altitude.

While previous studies using healthy rat models have shown that high altitude increases VPA absorption and MRT and decreasing its CL (Lin, 2022), the influence of disease state on drug metabolism remains to be fully elucidated. To better approximate clinical conditions, this study used the lithium chloride-pilocarpine method to establish an epileptic rat model, aiming to investigate the impact of high altitude on the PK of VPA in a more clinically relevant context. However, there were no statistical differences in C_max_ and t_1/2_ between the EV and EVH groups. C_max_ primarily reflects the drug absorption rate. The core alterations observed in this study were an increase in CL and a decrease in AUC of VPA in the EVH group, indicating that high altitude mainly affects the elimination rather than the absorption process of VPA. The apparent volume of distribution (V) in the EVH group also increased significantly. As t_1/2_ is determined jointly by CL and V, the effects of these two parameters are antagonistic: an increase in CL tends to shorten t_1/2_, while an increase in V tends to prolong it. This interplay between CL and V may explain why t_1/2_ showed only a trend toward shortening and did not reach statistical significance. Our results showed that the plasma concentration of VPA in rats from the EVH group was significantly lower than that in EV group at all time points, thus resulting in lower AUC. The CL was significantly accelerated and MRT was shortened, indicating that VPA was eliminated more rapidly from epileptic rats under high altitude environment. Therefore, the lack of statistical significance in C_max_ and t_1/2_ does not refute the core conclusion that high altitude significantly alters the PK of VPA, but rather reflects the differential effects of high altitude environment on various PK processes. This finding contrasts with previous studies and may be related to the characteristics of the epilepsy model used. This finding has important implications for drug therapy in epilepsy patients traveling to high-altitude areas.

Relying solely on blood drug concentrations as a surrogate indicator for central nervous system drug levels may lead to deviations in dosing regimens. Clinical observations indicated that some drug-resistant epilepsy patients fail to achieve expected therapeutic outcomes even when blood drug concentrations remain within the therapeutic window (Yoo and Panov, 2019). This may be related to the over-expression of efflux transporters such as P-glycoprotein (P-gp) in brain (Zhang et al., 2025). The blood-brain barrier (BBB) is primarily composed of outer cells, peripheral cells and astrocytes, and strictly controls the transport of molecules, proteins and particles into the brain (Kadry et al., 2020; Pedder et al., 2025). In the BBB, P-gp plays a critical role as a primary efflux transporter, actively pumping various drugs back into the bloodstream and thereby limiting their effective concentration in brain tissue (Paul et al., 2026; Shnayder et al., 2023). Paul et al. (2026) found that VPA is a substrate of P-gp at the BBB. High altitude environment significantly upregulate P-gp expression in the BBB of epileptic rats. This may be a key reason for the significant reduction in VPA concentration within the brain. In contrast, Lin et al. (2022) observed downregulation of P-gp expression in the BBB of healthy mice after 12 h of acute hypoxia. This discrepancy may be due to differences in animal models and hypoxia duration.

P-gp is expressed in the apical membrane of brain capillary endothelial cells that form the BBB, and its function is to limit brain entry of potentially cytotoxic compounds via active efflux into the blood (Noack et al., 2018). In a high altitude environment, upregulated P-gp expression reduces the amount of VPA crossing the BBB into the brain parenchyma, leading to decreased effective drug concentration in the brain. VPA exerts its antiepileptic effects in the brain through multiple mechanisms. On the one hand, it inhibits GABA transaminase, thereby increasing GABA levels in the brain to enhance inhibitory neurotransmission (Shnayder et al., 2023; Safdar and Ismail, 2023; Morland et al., 2018); on the other hand, it blocks voltage-gated sodium channels, stabilizing neuronal membrane potentials and suppressing abnormal high-frequency discharges (Shnayder et al., 2023; Safdar and Ismail, 2023). Elevated P-gp expression enhances VPA efflux in high altitude environment, resulting in insufficient VPA concentration in the brain. This weakens the above-mentioned inhibitory effect, lowers the seizure threshold, and ultimately reduces the antiepileptic efficacy of VPA.

However, the current evidence regarding whether P-gp is involved in VPA transport remains conflicting (Aylón Val and Hernando-Requejo, 2026). While Rawat et al. (2020) identified VPA as a P-gp substrate based on ATPase activity assays, Yang et al. (2021) provided data suggesting that VPA is a substrate for organic anion transporting polypeptide (OATP) family transporters, with no observable interaction with P-gp. Therefore, although multiple studies suggest that P-gp may participate in the efflux transport of VPA at the BBB, there remains controversy regarding which transporters are specifically responsible for mediating the cerebral distribution of VPA (Aylón Val and Hernando-Requejo, 2026; Baltes et al., 2007). For instance, some studies indicate that while VPA is not a direct substrate of ABCC2, the rs2273697 polymorphism of the ABCC2 gene can still significantly influence the plasma concentration of VPA in epilepsy patients (Chen et al., 2018). OATP2 has been identified as an important influx transporter for VPA at the BBB. Guo and Jiang (2016) demonstrated that OATP2 is functionally expressed in rat brain microvascular endothelial cells. They further showed that upregulation of OATP2 expression significantly increased cellular uptake of VPA, whereas knockdown of OATP2 using small interfering RNA markedly reduced VPA uptake. This finding directly demonstrated that OATP2 transports VPA across the BBB and that its expression level was positively correlated with cerebral uptake of VPA. Guo et al. (2021) further confirmed that OATP2 is a direct target of miR-23a-3p, and that its downregulation reduces VPA uptake. Additionally, Adkison and Shen (1996) demonstrated that a medium-chain fatty acid transporter (MCFAT) present at the rat BBB specifically mediates the cerebral uptake of valproate, whereas short-chain monocarboxylate transporters are not involved in this process. While these findings support the existence of carrier-mediated influx, they do not exclude the possibility that other transporters—such as MCT1—may contribute to valproate brain uptake under different physiological or developmental conditions (Vijay and Morris, 2014). Beyond transporters, VPA is primarily metabolized in the liver through three pathways: glucuronidation, mitochondrial β-oxidation, and the cytochrome P450 (CYP450) enzyme system (Methaneethorn, 2018; Yin and Li, 2024). The uridine diphosphate glucuronosyltransferase (UGT) isoforms involved in VPA metabolism include UGT1A3, 1A4, 1A6, 1A8, and 1A9 (Methaneethorn, 2018). The CYP450 enzymes mainly involved in its metabolism include CYP2C9, CYP2A6, and CYP2B6, among which CYP2C9 is the primary catalyst for CYP-mediated hydroxylation and desaturation, whereas the contributions of CYP2A6 and CYP2B6 are relatively minor (Monostory et al., 2019). High-altitude hypoxia may alter the expression or activity of key metabolizing enzymes (e.g., UGT isoforms (Kato et al., 2016) and CYP2C9 (Zhang et al., 2025)), thereby modifying VPA clearance and plasma concentrations, which in turn affects its entry into the brain. Collectively, alterations in these transporters and metabolizing enzymes under high altitude conditions may constitute key factors influencing the cerebral concentration of VPA.

Hypoxia-inducible factor 1α (HIF-1α) serves as the primary molecular mediator of the hypoxia response (Dai et al., 2024). Under hypoxia conditions, HIF-1α expression is upregulated, enabling it to dimerize with HIF-1β and bind to hypoxia response elements (HREs) in the promoter regions of various target genes, thereby activating numerous downstream genes (Dai et al., 2024; Wang et al., 2022). P-gp is one of the target genes of HIF-1α (Nath et al., 2025; Lu et al., 2016), possessing an HIF-1 binding site within its promoter region, thereby enabling HIF-1 to induce P-gp expression (Lu et al., 2016). This relationship was further supported by the finding that silencing HIF-1α significantly reduces P-gp expression, confirming P-gp as an HIF-1α–responsive gene (Li et al., 2022). Consistent with this mechanism, the present study observed a significant upregulation of both HIF-1α and P-gp expression in the BBB of epileptic rats exposed to high altitude environment. A substantial body of evidence has established a positive correlation between VPA dose, plasma concentration, brain concentration, and therapeutic efficacy (Semmes and Shen, 1991; Johannessen and Johannessen, 2003; Chadwick, 1985). Therefore, the reduced brain VPA exposure observed under high altitude conditions may be partially attributable to lower plasma VPA concentrations, in addition to P-gp-mediated efflux. The effects of VPA are mediated *via* central mechanisms following its penetration of the BBB (Löscher, 1999). Its mechanism primarily involves influencing central nervous system neurons to prevent or reduce pathological excessive discharges, or by elevating the excitability threshold of brain tissue to inhibit the spread of abnormal excitability, thereby eliminating or alleviating epileptic seizures (Löscher, 1999). Therefore, the high altitude environment appears to promote P-gp over-expression *via* HIF-1α upregulation, thereby enhancing the efflux of VPA across the BBB and ultimately reducing its effective concentration in brain tissue. Taken together, these results suggest that HIF-1α–mediated induction of P-gp, together with reduced systemic VPA exposure, represents key mechanisms by which high altitude exacerbates epileptic seizures through diminished central drug delivery.

Maintaining the structural and functional integrity of the BBB is critical to ensure a stable internal environment within the central nervous system (Huang et al., 2025). Certain types of epileptic seizures significantly disrupt the molecular integrity of the BBB, primarily through the downregulation of tight junction proteins (such as occludin, ZO-1 and claudin-8), ultimately leading to increased paracellular permeability (Castañeda-Cabral et al., 2020). Severe convulsive seizures and status epilepticus can directly compromise the integrity of the BBB and temporarily increase the transport of various substances from the bloodstream into the brain. Vascular endothelial growth factor (VEGF), a key pro-angiogenic factor, is significantly upregulated during epileptic seizures and promotes endothelial cell proliferation, migration, and neovascularization (Hoeben et al., 2004). The local hypoxia induced by epileptic seizures activates HIF-1α, which further accelerates angiogenesis by upregulating VEGF transcription. Meanwhile, VEGF also downregulates tight junction proteins, further compromising BBB integrity and increasing vascular permeability (Huang et al., 2025). Hypoxia itself can also enhance BBB permeability, and VEGF, as a key molecule regulated by hypoxia, can specifically bind to vascular endothelial cells and promote their growth, participating in hypoxia-induced vascular remodeling (Liu and Li, 2022). Therefore, high altitude hypoxia environment and the pathological damage caused by epilepsy may act together on the BBB, synergistically regulating the cerebral uptake and distribution of VPA by affecting tight junction protein expression and barrier permeability.

Based on the aforementioned pathophysiological background, we further analyzed the specific effects of high altitude environment on the cerebral distribution of VPA in epileptic rats. We found that plasma VPA concentrations decreased significantly in the EVH group compared to EV group, whereas the reduction in brain VPA concentrations was not proportional to the decrease in plasma levels. Meanwhile, the brain-to-plasma ratio was higher in the EVH group than in the EV group. This finding suggested that the effect of high altitude hypoxia on the distribution of VPA in the brain was not determined by a single factor, such as plasma concentration or P-gp-mediated efflux, but rather results from the combined action of multiple mechanisms. On the one hand, high altitude hypoxia can significantly reduce plasma VPA exposure by accelerating systemic drug clearance and increasing the apparent volume of distribution, thereby reducing its delivery to the brain. On the other hand, hypoxia induces compensatory cerebral vasodilation, increases cerebral blood flow (Carr et al., 2024; Ainslie et al., 2016; Mascarenhas et al., 2025), and enhances BBB permeability (Dunn and Isaacs, 2021; Halder et al., 2025). These compensatory mechanisms may moderately improve the efficiency of VPA transport into the brain, thereby partially counteracting the negative impact of reduced plasma exposure. This dynamic balance between negative factors (reduced plasma exposure) and positive factors (enhanced brain penetration) explains why the brain–plasma ratio was higher in the EVH group than in the EV group.

The brain has the highest oxygen consumption of all organs, which makes it particularly susceptible to oxidative stress (Lim and Thomas, 2020; Madireddy and Madireddy, 2023). Oxidative stress can increase neuronal excitability, thereby triggering epileptic seizures (Madireddy and Madireddy, 2023; Kaproń et al., 2020). Notably, oxidative stress is recognised as both the cause and consequence of epileptic seizures (Pearson-Smith and Patel, 2017). Current experimental evidence confirms the role of oxidative stress in the generation and progression of seizures (Łukawski and Czuczwar, 2023), and in mechanisms associated with drug resistance (Grewal et al., 2017). Numerous studies on humans and animals have also confirmed the link between oxidative stress and epilepsy (Kaproń et al., 2020; Dash et al., 2025). Reduced blood antioxidant status was observed in epilepsy patients (Wang et al., 2021). Patients with status epilepticus (SE) exhibited decreased activity of plasma SOD, GSH, and catalase (CAT), as well as diminished total serum antioxidant capacity (Kalita et al., 2019). Oxidative stress is characterised by an imbalance between the production of reactive oxygen species (ROS) and the body’s ability to repair the resulting damage (Dash et al., 2025). Exposure to high altitude hypoxia environment leads to reduced oxygen utilisation and increased production of ROS within brain tissue (Li et al., 2025). ROS can oxidize lipids, proteins, and DNA, causing damage to cellular structure and function (Zhang J. et al., 2024). The clearance of ROS relies on antioxidant systems, both enzymatic (e.g., SOD) and non-enzymatic (e.g., GSH) (Ildarabadi et al., 2024). We found that high altitude hypoxia environment significantly reduced GSH content and SOD activity in the brain tissue of epileptic rats. MDA is a lipid peroxidation metabolite. MDA levels serve as a reliable indicator of lipid peroxidation *in vivo* and indirectly reflect the extent of cellular damage. As a well-established biomarker, MDA is widely used to assess oxidative injury and to evaluate the efficacy of antioxidant interventions (Dönmezdil et al., 2016). As expected, MDA levels were significantly elevated in the brain tissue of epileptic rats exposed to high altitude conditions. Ramazi et al. (2020) also demonstrated that sinomenine significantly reduced seizure severity and incidence of SE, hippocampal aberrant MFS, and DNA fragmentation by restoring ROS, MDA, and SOD levels in the brain tissue of epileptic rats, while preventing neuronal density reduction.

Oxidative stress and inflammation play a crucial role in the development and progression of epilepsy (Ildarabadi et al., 2024). Oxidative stress can induce neuroinflammation and neurodegeneration before an epileptic seizure, thereby lowering the seizure threshold and promoting epilepsy (Ambrogini et al., 2019; Huang et al., 2018). Neuroinflammation has been observed in both animal models of epilepsy and in patients with epilepsy (de Zorzi et al., 2019; Dickstein et al., 2019). Increasing evidence suggests that inflammation contributes to the occurrence of both seizures and epilepsy (Siebenbrodt et al., 2022; Mukhtar, 2020). Studies demonstrate that recurrent epileptic seizures correlate with elevated levels of pro-inflammatory cytokines such as TNF-α, IL-1β, and IL-6 (Soltani et al., 2022; Aguilar-Castillo et al., 2024). IL-1β is a key inflammatory mediator in acute stress responses and tissue injury. Elevated IL-1β levels in the brain may increase inflammation within brain tissue, thereby triggering convulsions (Dundar et al., 2013). IL-1β also activates endothelial cells and neutrophils, enhances the expression of adhesion molecules and promotes the release of the pro-inflammatory factors IL-6 and IL-8 (Mao et al., 2017). Together with TNF-α, it induces various inflammatory responses, thereby triggering or exacerbating epileptic seizures (Mao et al., 2017). We examined the expression of inflammatory factors in brain tissue using epileptic rats as a research model. The results indicated that hypoxia environment significantly increases inflammatory damage to brain tissue. Heparin-modified superparamagnetic iron oxide nanoparticles (UFH-SPIONs) are stable, homogeneous nanosystems with antioxidant enzyme activity that can cross the BBB and become enriched in hippocampal epileptogenic foci. By reducing inflammatory responses and oxidative stress in hippocampal tissue, UFH-SPIONs can mitigate the severity of epileptic seizures (Xu et al., 2024). Coincidentally, piperine can alleviate neuronal inflammation and oxidative stress damage in the hippocampus, thereby producing beneficial effects in treating epilepsy (Mao et al., 2017). Therefore, the exacerbation of oxidative stress and inflammatory responses likely represents another key mechanism contributing to the diminished pharmacodynamic efficacy of VPA in high altitude environment.

## 5 Conclusion

In conclusion, high altitude environment not only exacerbate seizure severity but also significantly alter the PK and PD of VPA in epileptic rats. The diminished efficacy of VPA provides a direct rationale for adjusting its dosage in high altitude environment. This diminished efficacy is not only attributable to alterations in its PK behavior, but is also closely associated with factors exacerbated by the high altitude, such as increased oxidative stress, inflammatory responses, and altered expression of relevant transporter proteins. Based on the findings of this study, it may be advisable for epilepsy patients who are rapidly ascending to high altitude regions to appropriately increase their VPA dose, strengthen therapeutic drug monitoring and develop individualized dosing regimens according to seizure control, plasma drug concentrations and patient tolerance. However, this study was based on an epileptic rat model, and species differences are inevitable. Since the physiological responses of rats in high altitude differ from those of humans, the findings of this study cannot be directly extrapolated to clinical epilepsy patients. The specific extent to which the efficacy of VPA is reduced in high altitude environment, as well as safe and effective dosing regimens, still require further confirmation through high-quality clinical studies.

However, several limitations in the present study. First, rats were not subjected to perfusion or blood space correction before brain tissue collection. The measured brain VPA concentration may partly derive from residual intravascular drug rather than the actual concentration at the therapeutic target in the brain parenchyma. Consequently, the real effective concentration of VPA at its target site might be lower than the detected value. Second, the duration of high altitude exposure in rats was relatively short, which may not fully mimic the physiological status of epilepsy patients born or living at high altitude for many years. Therefore, the conclusions of this study are more applicable to epilepsy patients who rapidly migrate from plain to high altitude. In addition, due to species differences, the trough concentration of VPA in this study was lower than the clinical therapeutic range, and once-daily administration did not fully simulate the clinical dosing regimen, which limited the direct clinical translation of the results to a certain extent. Future studies should use long-term acclimatized animal models, adopt perfusion techniques, and use more frequent dosing regimens to further verify and expand our findings. More importantly, future clinical studies directly investigating indigenous or long-term resident populations at high altitude are warranted to comprehensively evaluate the impact of long-term physiological adaptations on the therapeutic efficacy of VPA.

## Data Availability

The original contributions presented in the study are included in the article, further inquiries can be directed to the corresponding authors.
